# No “Golden Hour” in Alaska: Characteristics and Implications of Medevac-centered Emergency Care

**DOI:** 10.5811/westjem.53174

**Published:** 2026-06-16

**Authors:** Brian Rice, Chelsea Williams, Cindy Y. Chang, Jeremy Wood, Henry Saul, Angelica Pritchard, Gretchen Day, Timothy Thomas, David Hao, Carla Britton, Robert Onders, Tina Hernandez-Boussard

**Affiliations:** * Stanford University, Department of Emergency Medicine, Palo Alto, California; † Maniilaq Association, Kotzebue, Alaska; ‡ University of Washington, Department of Emergency Medicine, Seattle, Washington; § Brigham and Women’s Hospital, Department of Emergency Medicine, Boston, Massachusetts; ¶ Alaska Native Tribal Health Consortium, Research Services, Anchorage, Alaska; || Harvard Medical School, Department of Emergency Medicine, Boston, Massachusetts; # Rural Provider Network, Alaska Native Tribal Health Consortium, Anchorage, Alaska; ** Alaska Native Epidemiology Center, Alaska Native Tribal Health Consortium, Anchorage, Alaska; †† Stanford University, Department of Biomedical Informatics Research, Palo Alto, California

## Abstract

**Introduction:**

Rural areas in the United States have higher mortality rates than urban regions, particularly from emergency care-sensitive conditions. Air medical ambulances (medevacs) are critical to emergency care access in the rural U.S., but limited data hinder the ability to study this critical system. In Alaska, medevac services often represent the sole method for connecting Alaska Native patients in remote clinics to physician emergency medical care. This study characterizes medevac use and timing between tribally administered village clinics and hospitals in Northwest Alaska. Our objective in this study was to describe the use and timing of medevacs and investigate how medevac decision-making impacts medevac transport times.

**Methods:**

We conducted a retrospective cohort analysis of medevac transfers between 2020–2024 from 11 Alaskan village clinics within the Maniilaq Association, which serves ~8,000 people over 35,862 square miles.We included 1,579 medevacs representing 1,119 unique patients flying from remote clinics to a central critical access hospital hub.. Our primary outcome measure was the association between short medevac decision times (< 60 minutes), and short total medevac times (< 180 minutes). We measured “flight time,” “decision time,” and “ground time” (time spent waiting for an aircraft to become available) as key components of total medevac time.

**Results:**

A total of 1,579 medevacs were deployed for predominantly Alaska Native (96.8%) patients, of whom 22.2% had traumatic injuries. Between 4.1–7.9% of community members in the region are transported by medevac each year. Median medevac time was 262 minutes (interquartile range [IQR] 196–402 [101–4,142]) from activation call to arrival at the critical access hospital. No medevacs were < 100 minutes. “Ground time” was the longest phase of medevac encounters overall (median ground time 105 minutes (IQR 81–187]) vs median decision time 75 minutes (IQR 42–130) or median flight time 44 minutes (IQR 35–77). We found no association between short medevac decision times and short medevac response time (P = .93). Medevac decision-making was the longest phase in only 6% of the shortest total medevacs (< 190 minutes), but it was the longest phase in 40% of the longest total medevacs ( > 400 minutes).

**Conclusion:**

We found four key themes: 1) no medevacs were < 100 minutes. Independent of human or system factors,atients cannot be transferred from a village clinic to a critical access hospital within the “golden hour” of emergency care; 2) short medevac decision times were not significantly associated with short overall medevac times; 3) the longest phase in a medevac transfer was “ground time”; and 4) in the subset of the longest total medevacs, medevac decision-making was much more likely to be the longest phase than in the shortest total medevacs. This emergency transfer system is not responsive to patient condition and suggests that medevac decision support may help for a subset of encounters.

## INTRODUCTION

Rural areas of the United States have higher age-adjusted mortality rates than urban areas, and this rural–urban gap has widened over the past two decades.^1^ When cause-specific mortality is studied, many of the causes with the greatest disparities, such as stroke and unintentional injuries, are emergency care-sensitive conditions whose outcomes depend on timely emergency care.^2^–^4^ Rural areas differ from urban areas in many ways affecting health and mortality outcomes including social determinants of health and healthcare access.^5^ Although 98.4% of people who live in the U.S. can reach an emergency department (ED) within 60 minutes, in Alaska, the largest and most rural state, only 80.7% can.^6^

This access gap reflects Alaska’s unique geography. The state averages 1.2 people per square mile across a landmass larger than California and Texas combined. Furthermore, over 80% of the state is not connected to a road system and consists of Alaska Native (AN) communities situated on ancestral lands connected primarily by airplanes.^7^ The AN people come from a legacy of strengths, and enormous cultural benefits come from living on traditional lands. Most of these communities have no health facilities staffed by physicians or nurses. Instead, primary, preventative, and emergency healthcare in rural Alaska is provided by the Alaska Tribal Health System (ATHS) using a “spoke and hub” system of village clinics and referral hospitals.^8^ The outlying “spoke” clinics are entirely staffed by non-physician clinicians (NPC), usually community health aide/practitioners (CHA/P).^9^ Without roads, private vehicles or ambulances cannot be used to connect patients at “spoke” clinics to physicians at “hub” facilities. Almost all emergencies within this off- the-road system require an air medical ambulance (medevac) to connect to higher levels of care. While it is known that response times for emergency medical services (EMS) can result in excess mortality, and that the majority of excess mortality in rural Alaska is associated with emergency conditions, the exact role of medevacs in this system has yet to be studied.^10^–^14^ EBSCO (Elton B. Stephens Company

Elsewhere in the U.S., medevacs are part of a larger EMS systems, predominantly using helicopters instead of ground ambulances for critical trauma patients.^15^–^18^ it still remains unknown which group of trauma patients might benefit most from HEMS rescue. Consequently, the unique aim of this study was to reveal which patients might benefit most from HEMS rescue. Methods Trauma patients (ISS ≥9 This builds on the “golden hour” for trauma rule, which stipulates that the first 60 minutes is most critical for patient survival; medevac time savings of just 17 minutes improve mortality.^19^,^20^ About 300,000 medevacs occur annually (~0.1% of the population); 30% are scene responses, and 70% are interfacility transports.^21^ both fixed- and rotor-wing. Transfers where then subcategorized into interfacility and scene responses. Frequencies for each category were generated and reported.\nRESULTS: A total of 317,267 air medical transfers were completed in 2021. These included 19,421 (6 %

**Population Health Research Capsule**

What do we already know about this issue?

*Little is known about the rural Alaska medevac system, which provides the only link to emergency care for communities in the state’s off-the-road system*.

What was the research question?

*What are the use patterns of medevacs in rural Alaska, and are short medevac decision times associated with short medevac response times*.

What was the major finding of the study?

*Annually, 4–8% of Alaskans were medevacked; none in < 100 minutes, and decision time < 60 min showed no association with total time of <180 min (P = .93)*.

How does this improve population health?

*By quantifying high use of a critical service that cannot respond faster to patient needs, findings redirect efforts to optimizing pre-medevac care and targeted decision support*.

By comparison, medevacs in rural Alaska are the sole emergency lifeline; no 9–1–1 services or ambulances exist in most villages. The CHA/P (or rarely nurse practitioner or physician assistant) who staff the clinics anchor frontline care and are trained to recognize emergencies. When they do, they contact a physician at one of six regional “hub” hospitals by phone or videoconference. Together they decide whether a patient can remain in the village, is stable enough to take the next available commercial flight to the nearest town with a critical access hospital to present to the ED there, or they require a medevac. Medevacs typically transport the patient from the village clinic to a critical access hospital ED. These critical access EDs are staffed 24 hours a day by family medicine and/or emergency physicians but lack surgical or specialty services. When specialty care (eg, pediatrics, general surgery, obstetrics, intensive care) is required, the patients receive medevacs to the tertiary referral center, the Alaska Native Medical Center (ANMC) in Anchorage. Only one previously published paper to date has looked at the use of medevacs within this spoke-and-hub model, but it did not contain patient-level detail.^22^

No prior work has quantified each medevac phase: *decision*, *ground*, and *flight* times. Decision time spans the patient’s arrival at a village clinic to medevac activation; ground time covers crew and aircraft preparation and may be extended by medevacs already engaged with other emergencies, duty-hour limits, and severe weather conditions; flight time is round-trip travel. Medevac teams (pilot plus two medics and/or nurses) can deliver advanced point-of care testing, airway support, blood products, and therapeutics including broad-spectrum antibiotics, vasopressors, and thrombolytics. All flying in Alaska carries an element of risk with the highest per capita crash rate in the U.S., and medevac accidents have proven disproportionately fatal.^23^,^24^

Studying each phase is essential for improving system quality. Medevac delays are so notorious that a recent Arctic emergency preparedness conference pronounced, “There is no golden hour in Alaska.”^25^ The challenges of linking clinic, flight, and hospital data has stymied patient-level analyses to date. Consequently, while medevac delays may be a modifiable mortality driver, without these data we cannot a) quantify time-loss bottlenecks, b) link operational delays to clinical outcomes, c) identify targets for quality improvement), or d) identify when to streamline medevacs vs bolster village care.

Our research provides the first patient-level analysis of medevac timing and utilization in rural Alaska. The ATHS provides all clinical care and accepts almost all transfers in these regions and, thus, represents an almost entirely closed system. The high quality, birth-to-death electronic health record (EHR) data for AN patients within this system create an environment uniquely suited for associating patient emergencies with long-term outcomes to better understand the impact of rural transport on emergency care.^26^ Generated from six years of collaboration between AN tribal health organizations and academic partners, these findings establish a foundational evidence base for developing protocols, enhancing training, implementing clinical decision support using machine-learning, and making systems improvements aimed at reducing mortality in rural AN emergency care.

## METHODS

### Study Design and Ethics and Tribal Review

This was a retrospective review of existing administrative, medevac, and EHR data. Ethical approval was obtained from the Alaska Area Institutional Review Board (IRB), with an IRB Reliance Agreement from Stanford University. Tribal approvals were received from the Maniilaq Association, Southcentral Foundation, and the Alaska Native Tribal Health Consortium (ANTHC).

### Study Site

The Maniilaq Association is a regional tribal health organization within the ATHS that represents 12 federally recognized Tribes in Northwest Alaska. They administer the Maniilaq Health Center, a 17-bed critical access hospital in Kotzebue, AK, and collaborate with the ANTHC that along with Southcentral Foundation help administer care at the ANMC in Anchorage. The Maniilaq Health Center has telemetry capacity and a five-bed emergency department (ED) serving a rural population of approximately 8,000 people. This facility has physicians and nurses, non-physician clinicians, obstetric midwives, dentistry, optometry, and behavioral health but no obstetricians, and no surgical or subspecialty services. Medevacs originate from 11 villages with a mean distance of 87 miles (range 42.4–155.3 miles) from the critical access ED, with no road access. Patients are seen in village clinics with limited treatment and testing capacity, staffed by one or more non-physician clinicians from 8 am–5 pm but with a non-physivcian clinician on call 24 hours a day, 365 days a year.

### Data Sources

We included all medevacs from Maniilaq Association village clinics to either a critical access or tertiary referral center ED from 2020–2024 in this study. We excluded rare, special-case destinations (eg, Providence Alaska Medical Center in Anchorage for specific cardiac emergencies). Medevacs were identified using internal administrative data. We then cross-referenced patients and dates with the Cerner EHR to link them to structured and unstructured EHR data. Medevac data available in the EHR were also exported and linked to each patient encounter. We obtained all patient characteristics from the Cerner EHR. Data were collected for each patient encounter in the window from seven days prior to medevac until 30 days post-medevac. We linked exported EHR data to administrative data using Python (Python Software Foundation, Wilmington, DE). For all 2023 patients, a physician reviewer manually extracted patient data. These data were used to error-check automated data extraction and linking. We used publicly available census, map, and shapefile data for location and map generation.

### Data Cleaning

We performed all data-cleaning in Python. Administrative data was extensively manually cleaned and normalized. The patient identifiers from these administrative data were cross-referenced against EHR records, which served as the “gold standard” for patient identifiers. Deterministic matching was followed by probabilistic matching using the *fuzzywuzzy* library requiring two or more matching identifiers (among name, dates of birth, and medical record numbers) to establish a match. If a two-identifier match could not be produced, the record was excluded from further analysis. The EHR-derived locations of service replaced manually entered data in all cases of disagreement. Times and dates of medevac activations and facility encounters were all obtained from EHR data and replaced manually entered dates in all cases of disagreement.

### Variables

We defined “medevac decision time” as the time from a patient encounter starting at the village clinic until the medevac activation event. This decision time was classified as “short” if a decision was made in < 60 minutes, and “long” if it took > 60 minutes. Because no standard definition exists for medevac decision- making, this definition was developed by consensus among three physicians involved in medevacs in the Maniilaq Association. “Flight time” was generated for each village using an average flight speed of 200 mph (appropriate for the craft typically flying in this region) and round-trip straight-line distances between villages and Kotzebue. “Ground time” was obtained by subtracting this flight time from the total time between medevac activation and the patient arriving at the receiving facility. Ground time was classified as “short” if it was < 180 minutes (defined using the upper limit of the interquartile range [IQR]), and “long” if it was > 180 minutes.

We extracted free-text chief complaints from free-text fields and classified them using a regular expressions approach that identified “obstetric” and “trauma” chief complaints and then labeled the remainder “medical/surgical.” Health insurance, race, sex, age, and vital signs were extracted from EHR. Cutoffs for hypoxia were oxygen saturation < 92%, a fever was defined as > 100.3 °F, and age-specific cutoffs were used for hypotension and tachypnea, and tachycardia for pediatric and adult patients.

### Data Analysis

Figures were produced in Python using *matplotlib* and maps using *geopandas*. Descriptive statistics (including means, standard deviations [SD], medians, IQR, and ranges) and comparative analysis was done in R (The R Foundation for Statistical Computing, Vienna, Austria). We analyzed categorical variables for significance using chi-squared tests. Continuous variables were analyzed for significance using *t*-test for means, Mann-Whitney for medians, Pearson correlation for linear correlation, and Spearman correlation for monotonic correlation. A threshold of 0.05 was set for significance.

## RESULTS

**Figure 1:**
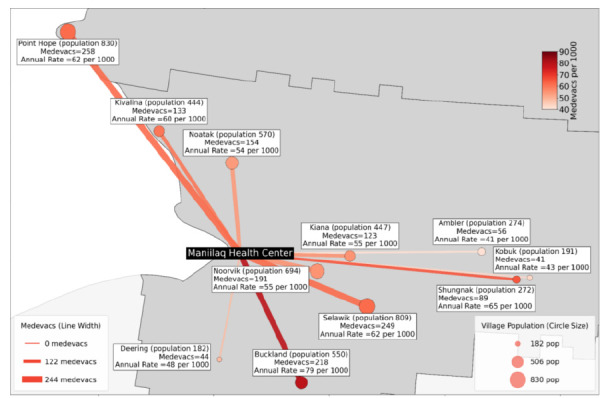
Map of medevacs within the Maniilaq Association, an Alaskan regional tribal health organization, in a study of medevac use and timing displaying village population, overall medevac use, and annual medevac rates per 1,000 persons, 2020–2024.

Overall, we located 2,670 records of medevacs for the Maniilaq Association from January 2020– December 2024. We found matching records between manual administrative and EHR data for 2,615 (97.9%) medevacs. Of this total, we included 1,579 in the analysis. The other 1,036 medevac interfacility transfers between critical access EDs and tertiary referral EDs were outside the scope of this study. Of the medevacs originating from village clinics, 1,257 (79.6%) had a medevac activation time, 1,350 (85.5%) had a village clinic encounter start time, and 1,460 (92.4%) had an ED arrival time. Medevacs went primarily to the critical access ED (97.1%, n = 1,535), with 44 patients (2.8%) bypassing the critical access ED and flying directly from the village to the tertiary referral center. The distribution of the patients flying to the critical access ED is shown as a map in Figure 1 Between 4.1–7.9% of the population of people living in rural villages in the Maniilaq Association receive a medevac each year (annual rates 41–79 per 1,000 persons). The characteristics of village clinic patients receiving a medevac are described in Table 1 below. These data show that the patients receiving medevac transport are overwhelmingly AN across a broad age range, and their primary payor is Medicaid. Most patients have at least one abnormal vital sign, and trauma accounts for only 22.2% of patients.

The median medevac was > four hours (262 minutes), and no patient transports occurred within one hour (minimum time 101 minutes). The longest medevac (4,142 minutes) took just under three days. A substantial minority of patients (39.1%) had a short medevac decision-making time of < 60 minutes. Most patients (64.0%) had a short medevac ground time of < three hours. Almost half the medevac activations (49.4%) occurred outside standard clinic operating hours.

Table 3 displays the association between medevac decision time and ground time. We found no association in the proportion of patients receiving a short medevac decision and a short ground time (chi-squared *P*-value = .93). Additionally, there was no significant difference in mean (*t*-test = 0.73) or median (Mann-Whitney = 0.72) ground time between short and long decision times, as well as no significant linear correlation (Pearson correlation *P* = .06) or monotonic correlation (Spearman correlation *P* = .93).

**Figure 2:**
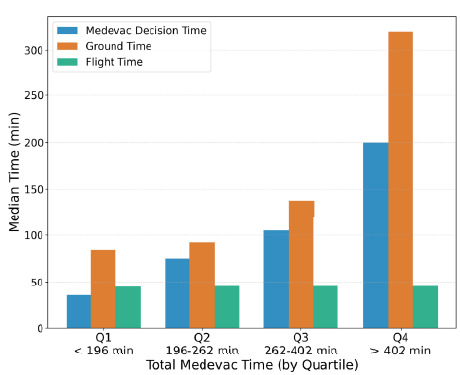
Bar plot of time spent in each phase of medevac process in a study of medevac use and timing, by quartiles of total medevac time (shortest to longest). *min*, minutes; *Q1-Q4*, quartiles 1–4.

Figure 2 illustrates medevac timing at a population level, grouping medevacs by quartiles of total time. Across all quartiles, median flight time remained consistent, and ground time represented the largest phase. Decision time was less than ground time in all quartiles, and both times increased monotonically across quartiles.

Table 4 describes the distribution of times for medevacs at the patient level. Overall, ground time was the longest phase for most (65.4%) patients. However, there was a sizable minority of patients (29.5%) for whom medevac decision was the longest phase. This proportion of patients increased for the longest two quartiles of patients, rising to 42.5% in Q3 and 39.9% in Q4.

## DISCUSSION

In this first published study of patient-level data for medevac transfers from rural Alaska, we uncovered important insights into the timing and utilization of air medical services in these remote areas. Our findings highlight the critical role that medevac services play in ensuring access to emergency care for rural Alaskan and especially AN communities. By examining the timing of medevac encounters, we provide a deeper understanding of the challenges faced by rural healthcare systems. This work identifies key areas for improvement in emergency response protocols and training, ultimately aiming to enhance the efficiency and effectiveness of village clinics and medevac services in rural settings.

This analysis highlights the central community health role of medevacs services. We report that 4.1–7.9% of all people living in these villages receive a medevac *each year* (Figure 1). This rate is 40–80 times higher than medevac rates in the continental U.S. but is similar to the 4.7% per capita annual EMS activation rate in rural America.^27^ While medevac is traditionally thought of as a high-resource, high-reward alternative for the sickest (typically trauma) patients, in rural Alaska it also fills a role similar to a standard ground ambulance as well as providing rapid access to high-level care for high-acuity patients. This underscores the major role that medevacs play in overall public health for these regions.

Without external comparators, optimal medevac rates remain uncertain. The next step is to link activations to ED dispositions and outcomes to identify who benefits from medevac and which incurs risk, displacement, and cost without clinical gain. Merging these data with regional and clinician decision-making trends, high-using patients, and local disease patterns can immediately inform policy and training. Furthermore, this next step can form the basis of clinical-decision support machine-learning models. We hope this overall research trajectory will empower long-term efforts to optimize use of this essential and limited resource and to improve emergency care outcomes for rural Alaskans.

The similarities between medevacs and ambulance EMS rates underscores the stark *dissimilarities* in the ability of these two systems to respond to critical patients (Table 3). Our study showed there was no association between fast medevac decisions and fast medevac responses. This highlights how medevacs in rural Alaska should be clearly understood to be interfacility transfers between clinic and ED rather than an extension of an EMS system. Conventional EMS coordinates multiple resources and prioritizes the critically ill with priority dispatch, running “lights and sirens” and – rarely – medevacs. In rural Alaska, the CHA/Ps provide de facto EMS by going to patient homes or accident scenes in the village but are outside the formal Alaska EMS system (although they do employ the skills of an emergency trauma technician or emergency medical technician that they receive in their training). However, true EMS services require components including staffing, training, communications, transportation, access to care, public safety agencies, standardized record-keeping, and system review and evaluation that do not exist in this context.^28^

The lack of association between decision and ground time reinforces the common regional understanding that medevac response times are largely independent and cannot respond to patient conditions. This critical finding has huge implications for public health policy. Medevacs are a critical but largely invariable tool. To reduce the time until a higher level of early emergency care can be provided, we must focus on the time prior to the medevac arrival. Opportunities exist to (a) expand the number of and integrate village non-physician clinicians into larger statewide EMS systems, and/or (b) expand the scope of care and resuscitation they can provide prior to medevac arrival. Combining this with expanding use of existing telehealth capacity for emergency care may help address the crisis of higher incidence of mortality related to emergency care and sensitive conditions in these regions.

The origins of the CHA/P program were rooted in the understanding that rural Alaska requires novel approaches and task-sharing to address rural care needs. When tuberculosis was the major issue 70 years ago, Alaska trained a novel cadre of paramedical professionals to expand rural care. Now, with a much-evolved system and increasing understanding of the needs for improved emergency care, it is possible to build on the CHA/P legacy of expanding the remote provision of care rather than following current trends in medical administration nationwide, which focus on the centralization of healthcare services.

Finally, our study characterizes total and phase-specific timing of medevac utilization. First, this study supports the adage that “There is no golden hour for trauma in Alaska,” as medevacs took hours or even days rather than minutes. No medevac response occurred in < one hour, with the shortest response times 101 minutes, the longest almost three days, and the median four hours and 22 minutes (Table 2). This underscores the need to strengthen village clinic-level care during these initial “golden hours.” Beyond total time, our analysis also showed that ground time exceeds decision time, and flight time is short and invariable (Figure 2). While some ground time is always required (preflight checks; moving crew and patients between plane and clinic), weather delays and aircraft/crew availability are the main driver of this longest time segment. While weather is uncontrollable, utilization is not and represents another target for quality improvement. Every medevac activation has downstream effects as duty-hour limits and single-mission capacity create queues that increase ground time for subsequent calls. Currently, medevac oversight rests with the hub physician and medevac dispatch and relies on their individual expertise and experience. Adding additional system-level metrics and decision support may improve medevac coordination and prioritization.

Decision time, while shorter than ground time, constitutes a large share as total time increases (Table 4). It is the longest phase for approximately 40% of the longest half of medevacs. This reflects the current clinical understanding that medevac patients are bimodal, including those with (a) immediately identifiable medevac needs, and (b) evolving needs identified after physician consultation, re-evaluations, and treatment response. This assessment process creates a median decision time of over three hours for the longest medevac encounters and presents a target for quality improvement. Standardized protocols and clinical decision support for non-physician clinicians and physicians could both expedite transfer decisions and identify patients who can safely remain in the village. Expanding village-based diagnostics, therapeutics, and telepresence/remote consultations may further optimize utilization at system and individual levels.

These findings highlight the need for targeted improvements in medevac protocols, training, and decision-making processes to enhance response times and overall patient outcomes. For community members, this research emphasizes the necessity of advocating for better healthcare resources and support systems. Developers and policymakers are encouraged to leverage these insights to create more effective emergency response frameworks that address the unique challenges of rural healthcare delivery in Alaska. By fostering collaboration among stakeholders, including clinicians, community organizations, and government agencies, we can work toward a more efficient and equitable emergency care system that ultimately saves lives and improves health outcomes in these underserved areas.

## LIMITATIONS

There are several limitations to this analysis. First, we use data from a single, critical-access hospital. Utilization may vary across other tribal hospitals, but since the structure of medevac services is similar across the ATHS, our findings are likely highly generalizable. This analysis includes medevac flights only and excludes commercial flights. This introduces potential selection bias as interventions targeting medevac efficiency may not address needs among patients who are transported by commercial aircraft. Medevac phases were extrapolated from facility EHR data rather than medevac EHR data. Flight times were derived using the average cruising speed of the aircraft used in the region rather than flight registers. Incorporating flight dispatch and medevac EHR data into future studies would improve timing accuracy. Finally, reliance on administrative data introduced human error with data entry. While extensive data-cleaning addressed this limitation, future studies would benefit from EHR adaptations to incorporate medevac activations as structured data fields.

## CONCLUSION

This study highlights the vital importance of medevac services in providing emergency care for rural Alaska and particularly the Alaska Native communities, representing a similar rate comparable to rural ambulances in the wider U.S. (4.1–7.9%). However, unlike traditional EMS systems, medevacs in the Alaska Tribal Health System are interfacility transports, and short decision times were not associated with short response times. Median medevac times were 4 hours, 22 minutes (range 101 minutes to ~3 days), reinforcing the absence of a “golden hour” for rural Alaskan emergency care. This evidence base lays the foundation for clinical decision support and health policy to reduce emergency care mortality in rural Alaska.

## Figures and Tables

**Table 1:** Village clinic medevac patient characteristics (n = 1,579) in a study of medevac use and timing in rural Alaska.

Characteristic	Value
% Female	52.8% (n = 834)
Age, mean (SD)	40.1 (25.2)
Race	
American Indian or Alaska Native	96.8% (n = 1,529)
Non-American Indian or Alaska Native	3.2% (n = 50)
Age Group (years)	
<5	8.6% (n = 136)
5–17	10.4% (n = 164)
18–40	37.3% (n = 589)
40–64	20.6% (n = 326)
65–80	15.8% (n = 250)
80+	7.2% (n = 114)
Insurance	
Medicaid	54.8% (n = 866)
Medicare	19.8% (n = 312)
Indian Health Service	12.9% (n = 204)
Commercial	11.0% (n = 173)
Other	1.5% (n = 24)
Vitals in Village Clinic	
All vitals normal	34.1% (n = 538)
Tachycardia	39.3% (n = 620)
Tachypnea	31.9% (n = 504)
Hypoxia	5.8% (n = 91)
Febrile	5.7% (n = 90)
Hypotension	2.0% (n = 31)
Chief complaint	
Medical/surgical	71.7% (n = 1,082)
Trauma/injury	22.2% (n =. 335)
Obstetric	6.1% (n = 92)

*SD*, standard deviation.

**Table 2:** Summary of medevac timing in a study of medevac use and timing in rural Alaska.

Characteristic	Value
Medevac timing	
Median decision time (clinic arrival to medevac activation) (min)	75 [IQR: 42–130, Range: 0–1057]
Median ground time (medevac activation to take off) (min)	105 [IQR: 81–187, Range: 23–4046]
Median calculated flight time (round trip from critical access ED to village and back) (min)	44 [IQR: 35–77, Range: 25–93]
Median total time (village clinic arrival to critical access ED arrival) (min)	262 [IQR: 196–402, Range: 101–4142]
Medevac decision time	
Short (0–60 min decision time)	39.1% (n = 369)
Long (≥ 60 min decision time)	60.9% (n = 574)
Medevac ground time	
Short ground time (≤ 180 min activate to takeoff)	64.0% (n = 774)
Long ground time (> 180 min activate to takeoff)	36.0% (n = 436)
Medevac total time (distribution across 4-hour categories)	
<4 hours	42.4% (n = 387)
4–8 hours	39.9% (n = 364)
8–12 hours	8.9% (n = 81)
12–16 hours	3.2% (n = 29)
≥16 hours	5.6% (n = 51)
Seasonal distribution	
Fall	26.9% (n = 424)
Spring	22.4% (n = 353)
Summer	25.6% (n = 405)
Winter	25.1% (n = 397)
Time of Day Distribution	
12 AM – 6 AM	19.2% (n = 259)
6 AM –12 PM	24.1% (n = 326)
12 PM – 6 PM	34.9% (n = 471)
6 PM – 12 AM	21.8% (n = 295)
Clinic open or closed	
Open (8 AM – 6 PM)	50.6% (n = 683)
Closed (5 PM – 8PM)	49.4% (n = 668)

*ED*, emergency department; *IQR*, interquartile range.

**Table 3:** Lack of association between medevac decision time and ground time in a study of medevac use and timing in rural Alaska.

Decision time	Short ground time (≤ 180 min activate to takeoff)	Long ground time (> 180 min activate to takeoff)	*P* value†
Short (0–60 min) decision time	74.0% (n = 267)	26.0% (n = 94)	.93
Long (> 60 min) decision time	73.7% (n = 406)	26.3% (n =145)	

† Chi-squared test used for significance.

*min*, minute.

**Table 4:** Time spent in each medevac phase in a study of medevac use and timing in rural Alaska.

Which medevac phase was the longest?	All Medevacs	Total time quartiles			

		Q1 (100–195 min)	Q2 (195–261 min)	Q3 (261–401 min)	Q4 (401–4141 min)
Medevac ground time	65.4% (n = 596)	87.3% (n = 199)	56.6% (n = 129)	57.5% (n = 131)	60.1% (n = 137)
Medevac decision time	29.5% (n = 269)	6.1% (n = 14)	29.4% (n = 67)	42.5% (n = 97)	39.9% (n = 91)
Flight time	5.2% (n = 47)	6.6% (n = 5)	14.0% (n = 32)	0.0% (n = 0)	0.0% (n = 0)

*min*, minutes; *Q*, quartile.
